# Predictors for Improvement of Mitral Regurgitation in Patients With Pure Severe Aortic Regurgitation Undergoing Transcatheter Aortic Valve Replacement: Can We Kill Two Birds With One Stone?

**DOI:** 10.31083/RCM47956

**Published:** 2026-06-08

**Authors:** Dawei Lin, Lifan Yang, Jianing Fan, Zilong Weng, Yuliang Long, Wenzhi Pan, Daxin Zhou, Junbo Ge

**Affiliations:** ^1^Department of Cardiology, Zhongshan Hospital, Fudan University, 200032 Shanghai, China; ^2^National Clinical Research Center for Interventional Medicine, 200032 Shanghai, China; ^3^Department of Cardiology, Shanghai Geriatric Medical Center, 201100 Shanghai, China

**Keywords:** mitral regurgitation, pure severe aortic regurgitation, transcatheter aortic valve replacement, predictors, logistic regression

## Abstract

**Background:**

Transcatheter aortic valve replacement (TAVR) is now recognized as an important treatment for pure severe aortic regurgitation (PSAR). Some patients with PSAR also suffer from functional mitral regurgitation (FMR). However, whether TAVR can improve FMR in patients with PSAR and the predictors of this improvement remain unknown. Thus, this study aimed to explore predictors of FMR improvement in patients with PSAR undergoing TAVR.

**Methods:**

Patients with PSAR and ≥mild FMR who underwent TAVR at the Zhongshan Hospital Affiliated with Fudan University were enrolled from June 2020 to June 2024. Participants were divided into groups with or without FMR improvement depending on whether FMR improved 1 month post-TAVR. Baseline data, imaging results, and follow-up data of the patients were collected.

**Result:**

This study included 111 patients, among whom 59 had improved FMR, and 52 did not. Compared to patients without FMR improvement, significantly fewer patients in the FMR-improved group were diagnosed with renal insufficiency (0% vs. 10%; *p* = 0.015) and left bundle branch block (0% vs. 8%; *p* = 0.030). Moreover, more were diagnosed with hypertension (80% vs. 56%; *p* = 0.007), and right bundle branch block (10% vs. 0%; *p* = 0.018). On transthoracic ultrasound, patients with FMR improvement were more likely to have a lower left ventricular ejection fraction (LVEF) and larger left ventricular end-diastolic (LVED) dimensions. In both groups, the degree of aortic regurgitation was significantly improved during 1-day and 1-month follow-ups post-TAVR (*p* < 0.001). No significant differences in the incidence of postoperative adverse events were found between the two groups during the short-term follow-up. Patients with higher degrees of FMR, lower LVEF, and hypertension were more likely to experience improvement in FMR post-TAVR.

**Conclusion:**

FMR improvement is observed in approximately half of PSAR patients undergoing TAVR. Higher FMR, lower LVEF, and hypertension before a TAVR are independent predictors of improvement in FMR.

## 1. Introduction

Aortic regurgitation (AR) is a common form of valvular heart disease that is characterized by the reflux of blood from the aorta into the left ventricle (LV) during diastole. Abnormalities within the aortic valve, aortic root, and ascending aorta contribute to AR [1]. The prevalence of AR in the population is estimated to be approximately 4.9%, with 0.5% of individuals experiencing moderate or greater AR [2, 3]. Surgical aortic valve replacement (SAVR) is the gold standard for treating pure severe aortic regurgitation (PSAR). Nevertheless, as many as one in five patients can only be treated conservatively because they are at high risk or have contraindications to surgery and have a higher mortality rate [4].

Over the past two decades, transcatheter aortic valve replacement (TAVR) has emerged as a less invasive alternative to surgery for the treatment of symptomatic severe aortic stenosis (AS), and its indications have gradually been extended to younger, low-risk patients with longer life expectancies [5, 6]. In recent years, TAVR has been increasingly used in patients with PSAR who have unfavorable risk profiles [7, 8]. Several meta-analyses have demonstrated the feasibility and safety of TAVR in treating selected PSAR patients who are unsuitable for SAVR [9, 10].

Mitral regurgitation is defined as retrograde systolic blood flow from the left ventricle to the left atrium, and can be independent or secondary to AR. Epidemiological data have found that moderate or severe regurgitation is the most common valvular disease in the United States [11] and the second most common valvular heart disease requiring surgery in Europe [4]. For AS patients treated with TAVR, significant mitral regurgitation (MR) at baseline and persistent MR after surgery is associated with an increased risk of all-cause mortality, cardiac death, and cardiac hospitalization [12, 13, 14]. However, the role of TAVR in treating patients with AR and MR remains unknown. Therefore, this study sought to investigate the safety and efficacy of TAVR in patients with AR and MR.

## 2. Subjects and Methods

### 2.1 Research Subjects

Patients who were diagnosed with PSAR and ≥mild functional mitral regurgitation (FMR), and underwent TAVR (Venus-A valve, Hangzhou QiMing Medical Equipment Co., Ltd.) at the Zhongshan Hospital Affiliated with Fudan University from June 2020 to June 2024 were enrolled. All patients were considered unsuitable for surgical valve replacement after a comprehensive evaluation. In addition, all patients had a follow-up of ≥1 month and underwent cardiac ultrasound examinations. The exclusion criteria were as follows: (1) patients with failed surgical bioprosthetic valves; (2) peak aortic valve pressure gradient measured by pre-TAVR echocardiography greater than 20 mmHg; (3) primary mitral regurgitation; (4) concomitant hypertrophic obstructive cardiomyopathy; and (5) concomitant left ventricular thrombus or infective endocarditis. This study was approved by the Ethics Committee of the the Zhongshan Hospital Affiliated with Fudan University (number: B2025-077), and all patients were informed and signed a consent form.

### 2.2 Patient Classification and Data Collection

Based on whether FMR improved post-TAVR, patients were divided into the MR-improved group and the MR-nonimproved group according to echocardiography. Clinical information on the patients was collected from medical records and the catheterization laboratory information system for retrospective analysis. The baseline information of patients, such as hypertension, diabetes, pulmonary arterial hypertension, atrial fibrillation, heart failure, and renal insufficiency before surgery, was documented. Patients underwent multidetector computed tomography (CT) and echocardiography examinations, and postoperative follow-up echocardiography was performed. Parameters such as left ventricular ejection fraction (LVEF), left atrial and left ventricular dimensions, degree of aortic valve regurgitation, and mean transvalvular pressure of the aortic valve were recorded. MR grading was established according to the European Society of Echocardiography diagnostic criteria [5]. It was quantified as none or trace, mild, mild-moderate, moderate, moderate-severe, or severe. Grading of AR severity was based on the valve regurgitant jet area obtained from echocardiography: none, trace, mild, moderate, and severe. FMR, which was improved post-TAVR, was defined as ≥1 degree reduction in MR degree by echocardiography at one month postoperatively.

### 2.3 Treatment and Follow-Up

All patients underwent TAVR via intravenous anesthesia by the structural heart disease surgery team in the Department of Cardiology, Zhongshan Hospital. When using Venus, an auto-expandible device to treat AR, the length from the base of the left coronary sinus to the midpoint of the anterior mitral leaflet should be greater than 25 mm; otherwise, the mitral valve function would be affected. Patients had a follow-up visit at the outpatient department 30 days postoperatively, at which time transthoracic echocardiography (TTE) and electrocardiography (ECG) examinations were performed and perioperative endpoint events recorded. The definition of clinical outcomes followed the Valve Academic Research Consortium-3 (VARC-3) criteria [12]. The primary endpoints included all-cause death and cardiovascular death. The secondary endpoints included TAVR-related complications, such as myocardial infarction, major bleeding events, major vascular complications, acute kidney injury, stroke, endocarditis, new-onset atrial fibrillation, implantation of a new pacemaker, coronary artery obstruction, moderate or greater paravalvular leak, and rehospitalization.

### 2.4 Statistical Analysis

Statistical analysis of the data was conducted using Stata 15.1 software (StataCorp LLC, College Station, TX, USA). The mean ± standard deviation (s) was used to represent normally distributed continuous variables, and proportions (%) were used for categorical variables. The comparison between the two groups was analyzed by an independent sample *t*-test and χ^2^ test. If the frequency was <5, Fisher’s exact probability method was used for comparisons between groups. A two-sided test was used. Univariable and multivariable logistic regression analyses were performed to evaluate the degree of correlation between MR improvement and clinical indicators. The quantitative data with a normal distribution among multiple groups were compared by analysis of variance, the quantitative data with a skewed distribution among multiple groups were compared by the Kruskal‒Wallis H test, the qualitative data between multiple groups were compared by the χ^2^ test, and the test level (α) was 0.05. Baseline characteristics showing significant differences between the two groups were first analyzed using univariate regression. Those with a *p*-value < 0.05 were then included in the multivariate regression model. In addition, several clinical factors with a strong correlation to mitral regurgitation (age, male, body mass index [BMI], left atrial diameter [LAD], and atrial fibrillation [AF]) were also incorporated into the univariate and multivariate regression analysis. Continuous variables in the model (age, BMI, LAD, left ventricle end-systolic dimension [LVEDs], and LVEF) were retained as continuous variables in the regression model.

## 3. Results

### 3.1 General Characteristics of Patients 

A total of 111 patients were enrolled, including 52 with persistent MR, with a mean age of 72.47 ± 7.12 years; 69 were males, and 42 were females (Table 1).

**Table 1.** **Comparison of baseline characteristics between the two groups**.

Patient characteristics	MR improved (n = 59)	MR nonimproved (n = 52)	*p* value
Age, years	73.29 ± 7.06	71.54 ± 7.15	0.198
Male, n (%)	39/59 (66%)	30/52 (58%)	0.362
Body mass index (kg/m^2^)	22.06 ± 4.09	21.71 ± 3.36	0.629
Smoke, n (%)	6/59 (10%)	6/52 (12%)	0.817
Hyperlipidemia, n (%)	17/59 (29%)	17/52 (33%)	0.658
Hypertension, n (%)	47/59 (80%)	29/52 (56%)	0.007
Diabetes mellitus, n (%)	4/59 (7%)	6/52 (12%)	0.382
Atrial fibrillation, n (%)	15/59 (25%)	10/52 (19%)	0.436
COPD, n (%)	5/59 (10%)	6/52 (10%)	0.922
Previous PCI, n (%)	2/59 (3%)	5/52 (10%)	0.178
PPM, n (%)	2/59 (3%)	3/52 (6%)	0.546
Peripheral vascular disease, n (%)	0/59 (0%)	2/52 (4%)	0.128
Renal insufficiency, n (%)	0/59 (0%)	5/52 (10%)	0.015
Left bundle branch block, n (%)	0/59 (0%)	4/52 (8%)	0.030
Right bundle branch block, n (%)	6/59 (10%)	0/52 (0%)	0.018
Atrioventricular block, n (%)	10/59 (17%)	7/52 (13%)	0.611
NYHA functional class III/IV, n (%)	52/59 (88%)	42/52 (81%)	0.282
Hemoglobin (g/L)	125.61 ± 16.54	124.40 ± 21.00	0.736
Serum creatinine (mg/dL)	112.70 ± 103.35	94.06 ± 34.83	0.218
NT-proBNP (pg/mL)	1569.86 ± 1591.81	1896.64 ± 2525.05	0.411
ALT (U/L)	27.27 ± 16.08	24.29 ± 12.93	0.327
STS risk score (%)	5.52 ± 1.90	5.51 ± 1.82	0.718

Abbreviations: MR, mitral regurgitation; COPD, chronic obstructive pulmonary disease; PCI, percutaneous coronary intervention; PPM, permanent pacemaker; NYHA, New York Heart Association; NT-proBNP, N-terminal pro-B-type natriuretic peptide; ALT, alanine aminotransferase; STS, Society of Thoracic Surgeons.

### 3.2 Comparison of Baseline Clinical Information and Imaging Data of Patients in the MR-Improved and MR-Nonimproved Groups 

Compared to patients without FMR improvement, significantly less patients in the FMR-improved group were diagnosed with renal insufficiency (0% vs. 10%, *p* = 0.015), left bundle branch block (0% vs. 8%, *p* = 0.030), and more were diagnosed with hypertension (80% vs. 56%, *p* = 0.007), and right bundle branch block (10% vs. 0%, *p* = 0.018). There were no significant differences in age, male sex, height, weight, body mass index, smoking, hyperlipidemia, diabetes mellitus, atrial fibrillation, previous percutaneous coronary intervention (PCI), permanent pacemaker (PPM), peripheral vascular disease, symptoms, atrioventricular block, New York Heart Association (NYHA) functional class III or IV, hemoglobin, serum creatinine, N-terminal pro-B-type natriuretic peptide, alanine aminotransferase (ALT), or Society of Thoracic Surgeons (STS) risk score between the two groups (Table 1). The TTE examination showed that patients in the MR-improved group had a lower ejection fraction (49.45 ± 8.05 vs. 60.53 ± 7.75, *p* < 0.001) and a larger LVEDs (41.91 ± 8.19 vs. 38.10 ± 6.96, *p* = 0.010). A greater proportion of patients in the MR-improved group had moderate or greater tricuspid regurgitation (TR) (22% vs. 5%, *p* = 0.014). No significant differences were found between these two groups in left atrial end dimension (LAED), left ventricle end-diastolic dimension (LVEDd), interventricular septal thickness (IVS), left ventricular outflow tract diameter (LVOTD), aortic valve peak velocity, mean valve gradient, effective orifice area, severe AR, moderate or greater TR, pulmonary artery systolic pressure (PASP), aortic annulus area, aortic annulus perimeter, or aortic annulus long axis diameter (Table 2).

**Table 2.** **Comparison of baseline imaging information between the two groups**.

			MR improved (n = 59)	MR nonimproved (n = 52)	*p* value
Echocardiography					
	LVEF (%)		49.45 ± 8.05	60.53 ± 7.75	<0.001
	LAED (mm)		44.21 ± 5.79	42.92 ± 5.69	0.332
	LVEDd (mm)		55.96 ± 7.40	54.29 ± 6.24	0.203
	LVEDs (mm)		41.91 ± 8.19	38.10 ± 6.96	0.010
	IVS (mm)		11.15 ± 1.68	10.84 ± 1.38	0.314
	LVOTD (mm)		30.56 ± 3.97	29.75 ± 3.25	0.317
	Aortic valve peak velocity (m/s)		2.14 ± 0.88	2.00 ± 0.65	0.370
	Mean valve gradient (mmHg)		10.9 ± 8.89	10.23 ± 7.05	0.931
	Effective orifice area (cm^2^)		2.73 ± 0.65	2.85 ± 0.65	0.321
	Severe AR, n (%)		59/59 (100%)	52/52 (100%)	-
	MR degree, n (%)				<0.001
		None or trace	0 (0%)	0 (0%)	
		Mild	10 (17%)	29 (56%)	
		Mild to moderate	16 (27%)	22 (42%)	
		Moderate	20 (34%)	1 (2%)	
		Moderate to severe	7 (12%)	0 (0%)	
		Severe	6 (10%)	0 (0%)	
	Moderate or greater TR, n (%)		11/59 (22%)	2/52 (5%)	0.014
	PASP (mmHg)		52.98 ± 10.19	52.25 ± 8.59	0.685
Computed tomography					
	Aortic annulus area (mm^2^)		533.12 ± 89.05	502.58 ± 80.00	0.106
	Aortic annulus perimeter (mm)		84.85 ± 7.45	82.56 ± 6.53	0.142
	Aortic annulus long axis diameter (mm)		28.87 ± 2.76	27.72 ± 2.69	0.060
	Largest diameter of ascending aorta (mm)		35.48 ± 5.73	35.99 ± 5.71	0.690

Abbreviations: LVEF, left ventricular ejection fraction; LAED, left atrial end dimension; LVEDd, left ventricular end-diastolic dimension; LVEDs, left ventricle end-systolic dimension; LVOTD, left ventricular outflow tract diameter; IVS, interventricular septal thickness; AR, aortic regurgitation; MR, mitral regurgitation; TR, tricuspid regurgitation; PASP, pulmonary artery systolic pressure.

### 3.3 Comparison of Preoperative and Postoperative Echocardiographic Follow-Up in the MR-Improved and MR-Nonimproved Groups

**Fig. 1.**
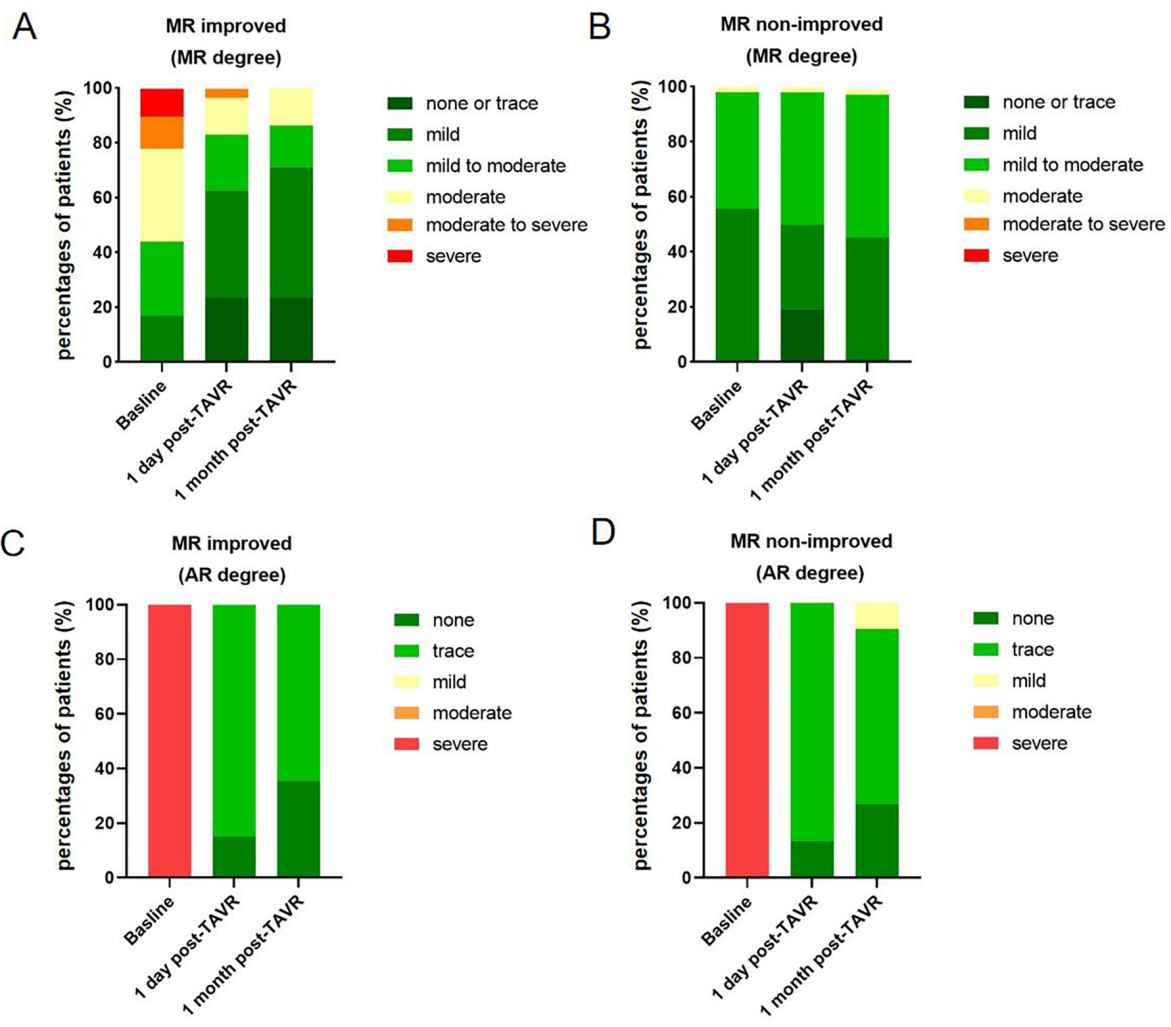
**Changes of AR and MR before and after TAVR were assessed in both groups**. (A) MR degree at baseline, 1-day post-TAVR, and 1-month post-TAVR in MR improved group. (B) MR degree at baseline, 1-day post-TAVR, and 1-month post-TAVR in MR non-improved group. (C) AR degree at baseline, 1-day post-TAVR, and 1-month post-TAVR in MR improved group. (D) AR degree at baseline, 1-day post-TAVR, and 1-month post-TAVR in MR non-improved group. MR, mitral regurgitation; AR, aortic regurgitation; TAVR, transcatheter aortic valve replacement.

**Fig. 2.**
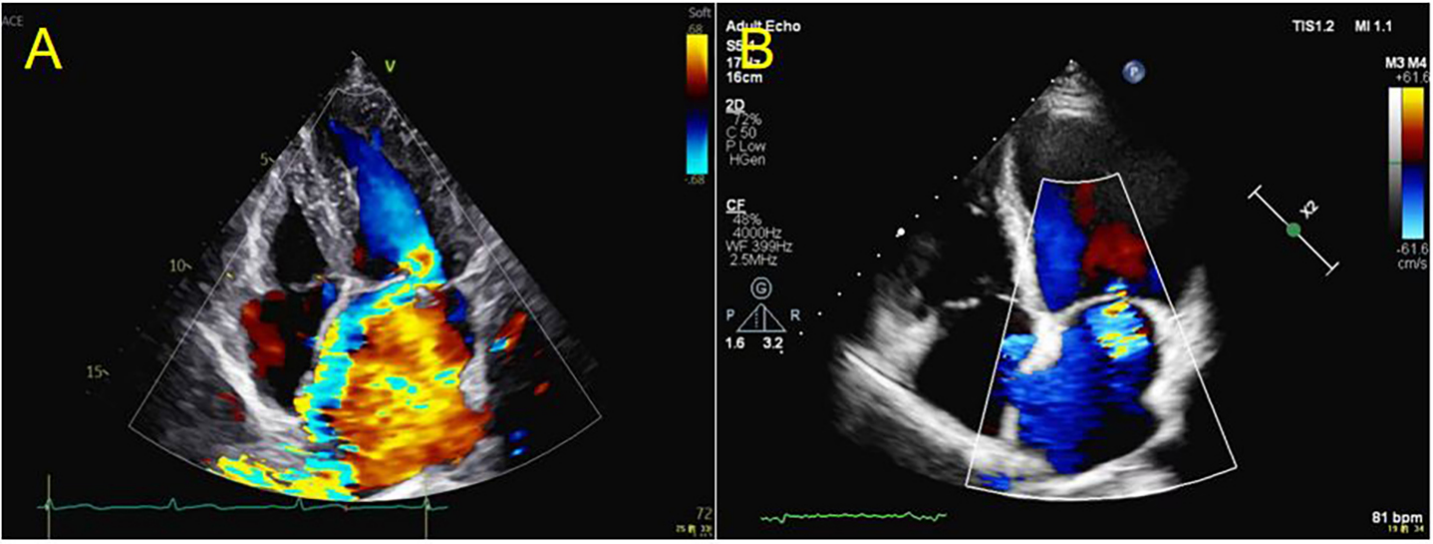
**The echocardiography examination showing the AR and MR before TAVR and 1-month post-TAVR**. (A) The echocardiography examination showed severe MR before TAVR. (B) The echocardiography examination showed trace MR one-month post-TAVR. AR, aortic regurgitation; MR, mitral regurgitation; TAVR, transcatheter aortic valve replacement.

The overall decrease in MR severity after TAVR was observed only in the MR-improvement group (preoperative vs. postoperative day 1 vs. postoperative month 1, *p* = 0.001), whereas postoperative AR severity significantly improved in both groups (preoperative vs. postoperative day 1 vs. postoperative month 1, *p* < 0.001) (Fig. 1A–D). Fig. 2 shows the AR and MR findings before TAVR and at 1-month post-TAVR on echocardiography.

### 3.4 Analysis of In-Hospital and Postoperative 1-Month Endpoint Events

There was no significant difference in primary and secondary endpoints in patients with or without improvement of MR who underwent TAVR in the hospital and 1 month after the operation. All-cause death or cardiac death did not occur in the two groups. During hospitalization, bleeding events, major vascular complications, stroke, new-onset atrial fibrillation, new-onset left bundle branch block, new-onset atrioventricular block, permanent pacemaker implants and moderate-to-severe paravalvular leaks were 0%, 2%, 0%, 11%, 19%, 18%, 19%, and 11% in the MR improved group and 4%, 2%, 4%, 13%, 12%, 23%, 13%, and 13% in the MR nonimproved group, respectively. In the 1-month follow-up, 2% stroke, 2% new-onset atrioventricular block, 2% new permanent pacemaker implantation, 8% paravalvular leak (PVL), and 4% rehospitalization were observed in the MR-improved group. The non-improved MR group had 4% bleeding events, 2% stroke, 4% new left bundle block, 2% new-onset atrioventricular block, 2% permanent pacemaker, 7% moderate or greater paravalvular leaks, and 9% rehospitalization (Table 3).

**Table 3.** **Comparison of clinical endpoints at different follow-up time points between the two**.

Clinical end-points, n (%)		In-hospital			1-month		
		MR improved (n = 59)	MR nonimproved (n = 52)	*p*	MR improved (n = 59)	MR nonimproved (n = 52)	*p*
Primary endpoints							
	All-cause mortality	0 (0%)	0 (0%)	-	0 (0%)	0 (0%)	-
	Cardiovascular mortality	0 (0%)	0 (0%)	-	0 (0%)	0 (0%)	-
Secondary endpoints							
	Bleeding event	0 (0%)	2 (4%)	0.180	0 (0%)	2 (4%)	0.180
	Major vascular complication	1 (2%)	1 (2%)	0.928	0 (0%)	0 (0%)	-
	Acute renal failure	0 (0%)	0 (0%)	-	0 (0%)	0 (0%)	-
	Stroke	0 (0%)	2 (4%)	0.180	1 (2%)	1 (2%)	0.928
	Myocardial infarction	0 (0%)	0 (0%)	-	0 (0%)	0 (0%)	-
	New AF	6 (11%)	6 (13%)	0.817	0 (0%)	0 (0%)	-
	New LBBB	9 (19%)	7 (12%)	0.788	0 (0%)	2 (4%)	0.180
	New AVB	10 (18%)	11 (23%)	0.572	1 (2%)	1 (2%)	0.928
	New PPM	11 (19%)	6 (13%)	0.300	1 (2%)	1 (2%)	0.928
	Endocarditis	0 (0%)	0 (0%)	-	0 (0%)	0 (0%)	-
	Perivalvular leakage	6 (11%)	6 (13%)	0.817	4 (8%)	3 (7%)	0.827
	Rehospitalization	0 (0%)	0 (0%)	-	2 (4%)	4 (9%)	0.317

Abbreviations: AF, atrial fibrillation; LBBB, left bundle branch block; AVB, atrioventricular block; PPM, permanent pacemaker; PVL, paravalvular leak.

### 3.5 Univariable and Multivariable Logistic Regression Analyses for FMR Improvement in PSAR Patients Post-TAVR 

The results of univariable and multivariable logistic regression analyses for the FMR improvement are shown in Table 4. Age, male, BMI, LAD, and AF were incorporated into the univariable and multivariable logistic regression analysis. Among the variables, MR degree, LVEDs, hypertension, and LVEF were associated with FMR improvement (all *p* < 0.05). In the multivariable analysis, higher MR degree, lower LVEF, and suffering from hypertension were independently associated with FMR improvement in patients post-TAVR (Table 4).

**Table 4.** **Logistic regression**.

Variables	Univariate analysis			Multivariate analysis		
	OR	95% CI	*p*	OR	95% CI	*p*
Age	1.036	0.982–1.093	0.198	0.967	0.880–1.061	0.476
Male	1.805	0.834–3.904	0.134	0.410	0.107–1.574	0.194
BMI	1.043	0.941–1.155	0.425	1.047	0.869–1.261	0.631
MR degree	1.753	1.190–2.582	0.004	4.361	1.835–10.365	0.001
LVEDs	1.054	1.003–1.109	0.038	0.980	0.901–1.067	0.647
Hypertension	2.597	1.136–5.925	0.024	3.950	1.007–15.489	0.049
LVEF	0.959	0.921–0.999	0.046	0.882	0.814–0.955	0.002
LAD	0.990	0.924–1.060	0.766	0.983	0.876–1.104	0.777
AF	1.432	0.579–3.539	0.437	0.867	0.196–3.823	0.850

Abbreviations: CI, confidence interval; BMI, body mass index; MR, mitral regurgitation; LVEDs, left ventricular end-systolic dimension; LVEF, left ventricular ejection fraction; LAD, left atrial diameter; AF, atrial fibrillation.

## 4. Discussion

This study investigated the improvement in FMR post-TAVR in patients with PSAR. The key findings are as follows: (1) FMR in more than half of the patients (53.15%) improved with TAVR for PSAR. (2) A higher FMR degree, lower LVEF, and hypertension before TAVR are independent predictors of FMR improvement. (3) No significant differences were found in the incidence of postoperative adverse events between the two groups during the short-term follow-up.

In our study, with a limited sample size, the prevalence of FMR in PSAR patients is up to 45%. The reflux of blood and subsequent increased back pressure lead to significant volume overload, resulting in significant MR. Yang *et al*. [15] found that patients with both significant AR and secondary MR resulted in a 2.34-fold increase in risk for mortality relative to the expected survival of an age- and sex-matched population, while the group with pure AR only had a 1.25-fold excess mortality risk. In addition, patients with residual mild MR after mitral valve repair have an increased risk of early adverse outcomes [16, 17, 18]. However, the best therapy for that group of patients remains undecided [19]. Some studies recommend SAVR because MR and AR can be treated simultaneously [19]. However, surgery would not be suitable for some patients, especially those with higher STS scores or elderly patients. In contrast to SAVR, patients who undergo TAVR do not routinely undergo concomitant interventions on the mitral valve, even if significant MR is present at the time of the procedure. Therefore, we explored whether TAVR could improve not only AR but also FMR. The present study found that 53.15% of patients were observed to have an improvement in FMR via TAVR for PSAR. Therefore, for those patients with PSAR who would have an improvement in FMR post-TAVR, TAVR can be considered an effective strategy for PSAR, which can kill two birds with one stone.

We found that patients with lower LVEF and hypertension are more likely to have improved FMR post-TAVR. The underlying mechanisms may be as follows: First, hypertension increases the load on the aortic valve, inducing AR, subsequently increasing left ventricular pressure, and leading to greater pressure on the mitral valve, leading to FMR. After the AR improved, the pressure on the mitral valve decreased, and the FMR improved. Second, a higher degree of FMR indicates the existence of increased mitral valve transvalvular gradients and more reflux. Therefore, when regurgitation was decreased, the change in MR reflux was greater in the higher-level FMR degree group than in the lower-level FMR group. Third, a higher level of AR at baseline would result in more blood retention in the left ventricle, which leads to decreased LVEF over time and causes the left ventricle to become larger, which leads to increased FMR. This study is the first report to explore the predictors of MR improvement post-TAVR for PSAR. A previous study investigated the predictors of MR improvement after TAVR for AS, and ejection fraction and LVED were found to be predictors [20]. Our study did not distinguish between atrial FMR and ventricular FMR, which may have different responses to TAVR. Therefore, the classification of FMR needs to be considered when conducting further studies.

During the 1-month follow-up, no major adverse events were found in either group. The results demonstrated that it is safe and efficient for PSAR patients with mild to severe FMR to undergo TAVR. Zheng *et al*. [21] also demonstrated the safety and efficacy of transfemoral TAVR with the Venus A-Valve in the treatment of patients with AR. In addition, no significant differences were found in the incidence of postoperative adverse events between the MR improved group and the non-MR improved group during the short-term follow-up. However, we did not have a longer follow-up period, and the results may be different with a longer follow-up period. Mavromatis *et al*. [22] found that, in contrast with the MR improved group, the patients without MR improvement undergoing TAVR for AS had an increased mortality and the need for heart failure rehospitalization.

### Limitation

This study also has some limitations. First, the lack of CT parameters for FMR restricted our further investigation on tenting height, valve leaflet motion, and mitral calcification, which should be taken into consideration in future studies. Second, the number of patients in our study is small, and more patients should be enrolled in future research. Third, further screening and study of the population with the presence of ≥ moderate MR after TAVR should be followed. Finally, the follow-up time was limited, and the results should be further confirmed by future studies with larger sample sizes and prolonged follow-up.

## 5. Conclusion

FMR improvement is observed in approximately half of PSAR patients undergoing TAVR. A higher degree of FMR, lower LVEF, and hypertension pre-TAVR are independent predictors. TAVR appears to be a safe and efficient treatment for patients with simultaneous PSAR and FMR.

## Data Availability

All data generated or analyzed during this study are included in this published article.
